# Lymphoma-Sink Effect in Marginal Zone Lymphoma Based on CXCR4-Targeted Molecular Imaging

**DOI:** 10.1007/s11307-023-01830-9

**Published:** 2023-06-07

**Authors:** Aleksander Kosmala, Simone Seifert, Simone Schneid, Niklas Dreher, Takahiro Higuchi, Alexander Weich, Sebastian E. Serfling, Philipp E. Hartrampf, Hermann Einsele, Andreas K. Buck, Max S. Topp, Johannes Duell, Rudolf A. Werner

**Affiliations:** 1 Department of Nuclear Medicine, University Hospital Würzburg, Oberdürrbacher Strasse 6, 97080 Würzburg, Germany; 2 Faculty of Medicine, Dentistry and Pharmaceutical Sciences, Okayama University, Okayama, Japan; 3 Department of Internal Medicine II, University Hospital Würzburg, Würzburg, Germany; 4 Johns Hopkins School of Medicine, The Russell H Morgan Department of Radiology and Radiological Sciences, Baltimore, MD USA

**Keywords:** CXCR4, C-X-C motif chemokine receptor, PET, [^68^Ga]Ga-PentixaFor, Theranostics, Radioligand therapy, Tumor sink, Lymphoma sink

## Abstract

**Purpose:**

Recent studies investigating a tumor-sink effect in solid tumors reported on decreasing uptake in normal organs in patients with higher tumor burden. This phenomenon, however, has not been evaluated yet for theranostic radiotracers applied to hematological neoplasms. As such, we aimed to determine a potential “lymphoma-sink effect” in patients with marginal zone lymphoma (MZL) imaged with C-X-C motif chemokine receptor (CXCR) 4-directed PET/CTs.

**Procedures:**

We retrospectively analyzed 73 patients with MZL who underwent CXCR4-directed [^68^Ga]Ga-PentixaFor PET/CT. Normal unaffected organ uptake (heart, liver, spleen, bone marrow, kidneys) was quantified using volumes of interests (VOIs) and mean standardized uptake values (SUV_mean_) were derived. MZL manifestations were also segmented to determine the maximum and peak standardized uptake values SUV (SUV_max/peak_) and volumetric parameters, including lymphoma volume (LV), and fractional lymphoma activity (FLA, defined as LV*SUV_mean_ of lymphoma burden). This approach resulted in 666 VOIs to capture the entire MZL manifestation load. We used Spearman’s rank correlations to determine associations between organ uptake and CXCR4-expressing lymphoma lesions.

**Results:**

We recorded the following median SUV_mean_ in normal organs: heart, 1.82 (range, 0.78–4.11); liver, 1.35 (range, 0.72–2.99); bone marrow, 2.36 (range, 1.12–4.83); kidneys, 3.04 (range, 2.01–6.37); spleen, 5.79 (range, 2.07–10.5). No relevant associations between organ radiotracer uptake and MZL manifestation were observed, neither for SUV_max_ (ρ ≤ 0.21, *P* ≥ 0.07), SUV_peak_ (ρ ≤ 0.20, *P* ≥ 0.09), LV (ρ ≤ 0.13, *P* ≥ 0.27), nor FLA (ρ ≤ 0.15, *P* ≥ 0.33).

**Conclusions:**

Investigating a lymphoma-sink effect in patients with hematological neoplasms, we observed no relevant associations between lymphoma burden and uptake in normal organs. Those observations may have therapeutic implications, e.g., for “cold” SDF1-pathway disrupting or “hot,” CXCR4-directed radiolabeled drugs, as with higher lymphoma load, normal organ uptake seems to remain stable.

**Supplementary Information:**

The online version contains supplementary material available at 10.1007/s11307-023-01830-9.

## Introduction

C-X-C motif chemokine receptor 4 and its only known ligand SDF1 are crucially involved in stem cell mobilization and migration of CXCR4-positive bone marrow (BM) cells by following a gradient towards (neo-)angiogenic niches, finally leading to promotion of tumor-feeding vessels and metastatic spread [1]. For instance, in specimen of patients afflicted with marginal zone lymphoma (MZL), more than 90% revealed relevant CXCR4 expression and those *ex-vivo* findings then laid the proper groundwork for *in-vivo* imaging using CXCR4-directed [^68^Ga]Ga-PentixaFor PET/CT [2, 3]. Further favoring a more wide-spread adoption in hemato-oncology, hematological neoplasms achieved substantially higher target-to-background ratios and maximum standardized uptake values (SUV_max_) on a quantitative level when compared to most solid tumor entities [4]. As such, beyond enabling a more precise diagnostic read-out, CXCR4-targeted PET/CT may also allow to identify candidates for treatment with ß^-^(minus)-emitting counterparts, e.g., [^177^Lu]Lu-/[^90^Y]Y-PentixaTher [5]. Of note, such a molecular imaging-based treatment strategy may then achieve both anti-lymphoma and desired myeloablative effects, thereby preparing for subsequent hematopoietic stem cell transplantation (HSCT) in addition to conditioning regimens [6, 7].

Increasing tumor load, however, may be linked to decreasing radiotracer accumulation in normal organs, possibly explained by a lower amount of tracer available to the normal organs in the presence of high tumor load. Such a tumor-sink effect has been described for solid tumors including prostate carcinoma (PC) imaged with prostate-specific membrane antigen (PSMA)-targeted PET [8]. A recent evaluation using chemokine receptor imaging in patients diagnosed with other solid tumor entities than PC did not reveal such an interdependence on an organ-tumor level [9]. For hematological neoplasms, a potential “lymphoma-sink” effect has been shown in patients with diffuse large B cell lymphoma (DLBCL) imaged with 2-[^18^F]FDG PET/CT [10, 11]. For CXCR4-targeted PET/CT, however, a lymphoma-sink effect has not been determined yet. In a theranostic setting, those information may be of relevance, as in patients with increasing lymphoma load, higher [^177^Lu]Lu-/[^90^Y]Y-PentixaTher activities could then be administered, thereby optimizing anti-lymphoma efficacy and minimizing side effects in organs at risk [6, 12]. As such, investigating the largest cohort of MZL patients imaged with [^68^Ga]Ga-PentixaFor to date, we aimed to investigate whether such a potential lymphoma-sink effect is also present in individuals with MZL scheduled for chemokine receptor PET.

## Methods

We retrospectively identified 73 patients with MZL who underwent [^68^Ga]Ga-PentixaFor PET/CT from our institutional PET/CT database. The local institutional review board waived the need for further approval due to the retrospective nature of this study (waiver no° 20210726 02). Written informed consent of all patients was collected beforehand. Parts of this patient cohort have been investigated in previous studies [3, 4], however, without examining potential implications of lymphoma burden on normal organ radiotracer uptake.

### Imaging Procedure

Biograph mCT64 or 128 (Siemens Healthineers, Erlangen, Germany) PET/CTs were used for [^68^Ga]Ga-PentixaFor exams. The scans raged from vertex of the skull to the proximal thighs. A mean of 128 MBq (± 24.5 MBq) of [^68^Ga]Ga-PentixaFor was applied and images were acquired approximately 1 h after injection. PET/CT scans were acquired in 3D mode with an acquisition time of 2 min/bed position (mCT64) or continuous bed motion with flow bed velocity of 1.1 mm/s (mCT128). For image reconstruction, Gaussian filter was set to 2.0 mm using a matrix of 200*200 at 3 iterations with 24/21 subsets (mCT 64/mCT 128), using point spread function/time of flight (mCT 64/mCT 128). CT scans with or without contrast enhancement were used for attenuation correction [3, 9].

### Image Analysis

Images were analyzed on a dedicated workstation using syngo.via software (version VB60A; Siemens Healthineers, Erlangen, Germany). [^68^Ga]Ga-PentixaFor normal organ uptake was established by one reader (S.Sc.) by placing spherical volumes of interest (VOI) with a minimum diameter of 10 mm as previously described [9, 13–15]. To determine mean standardized uptake values (SUV_mean_) for normal unaffected organs, in each patient a sum of 8 VOIs in the liver, spleen, lateral myocardial wall of the left ventricle, both kidneys, and the vertebral bodies of C2, Th7, and L5 was drawn [9, 13–15]. For mean kidney and BM uptake, the average of the respective organ’s VOIs was considered. To account for potential bone marrow involvement [16], the bone marrow was considered an unaffected normal organ only in patients with negative bone marrow biopsy results within 30 days of CXCR4-directed PET/CT. In addition, VOIs were also placed completely covering each MZL manifestation showing radiotracer accumulation above background levels, which automatically generated a 3-dimensional VOI at a 40% isocontour threshold. Inconclusive findings were verified by expert readers (S.Se., R.A.W., A.K.). Different quantitative aspects of total MZL load were derived, including averaged maximum (SUV_max_), peak (SUV_peak_, defined as average SUV within a 1cm^3^ sphere around the hottest voxel) and SUV_mean_, sum of lymphoma volume (LV in cm^3^), and sum of fractional lymphoma activity (FLA, defined as lymphoma SUV_mean_*LV) [9, 13–15]. MZL manifestations smaller than 15 mm or 1.7 cm^3^ were not sampled to account for potential partial volume effects [13, 14, 17].

### Statistical Analysis

GraphPad Prism version 9.3.1 (GraphPad Prism Software, La Jolla, CA, USA) was utilized for statistical analyses. Gaussian distribution was determined using the Shapiro-Wilk test. We used Spearman’s rank correlation coefficient (ρ) for correlative analyses [9, 13]. *P* < 0.05 was considered statistically significant.

## Results

### Patient Population

Mean age of the entire cohort was 66.2 ± 12.3 years and 40/73 (54.8%) were female. The most common MZL subtype was extranodal (44/73, 60.3%), followed by nodal (24/73, 32.9%) and splenic MZL (5/78, 6.8%; Table 1).

**Table 1:** Patients’ details

Age^†^		66.2 ± 12.3 years
Gender (female)		40/73 (54.8%)
MZL subtype	Extranodal	44/73 (60.3%)
	Nodal	24/73 (32.9%)
	Splenic	5/73 (6.8%)
Prior therapies	In total	6/73 (8.2%)
	Surgery	2/6 (33.3%)
	Chemotherapy	2/6 (33.3%)
	Radiation therapy	3/6 (50.0%)

^†^Values are mean ± standard deviation

### Normal Organ Radiotracer Uptake and Lymphoma Manifestation Load

Table 2 shows descriptive statistics of normal organ radiotracer uptake and lymphoma load. In the 5 patients with splenic MZL subtype, no normal organ VOIs were placed in the spleen. In 31 patients, bone marrow infiltration could not be ruled out by concurrent bone marrow biopsy results. Consequently, a total of 8 * 73–5–(31 * 3) = 486 VOIs in unaffected organs was analyzed. For lymphoma load, a total of 666 VOIs was drawn (median, 5; range, 1–88), resulting in an overall number of 486 + 666 = 1152 VOIs.

**Table 2:** Descriptive statistics of normal organ uptake and lymphoma manifestation burden

		Parameter	Minimum	Median	Maximum
Normal Organs	Heart	SUV_mean_	0.78	1.82	4.11
	BM	SUV_mean_	1.12	2.36	4.83
	Liver	SUV_mean_	0.72	1.35	2.99
	Spleen	SUV_mean_	2.07	5.79	10.5
	Kidneys	SUV_mean_	2.01	3.04	6.37
Lymphoma Burden		SUV_max_	2.53	9.37	29.5
		SUV_peak_	1.58	5.31	20.2
		LV	1.74	24.2	934
		FLA	9.78	176	7522

*BM* bone marrow, *SUV*_*mean*_ mean standardized uptake value, *SUV*_*max*_ maximum standardized uptake value, *SUV*_*peak*_ peak standardized uptake value, *LV* lymphoma volume (in cm^3^), *FLA* fractional lymphoma activity (defined as mean standardized uptake value*LV)

### Correlative Analyses of Radiotracer Uptake and Lymphoma Manifestation Load

**Fig. 1:**
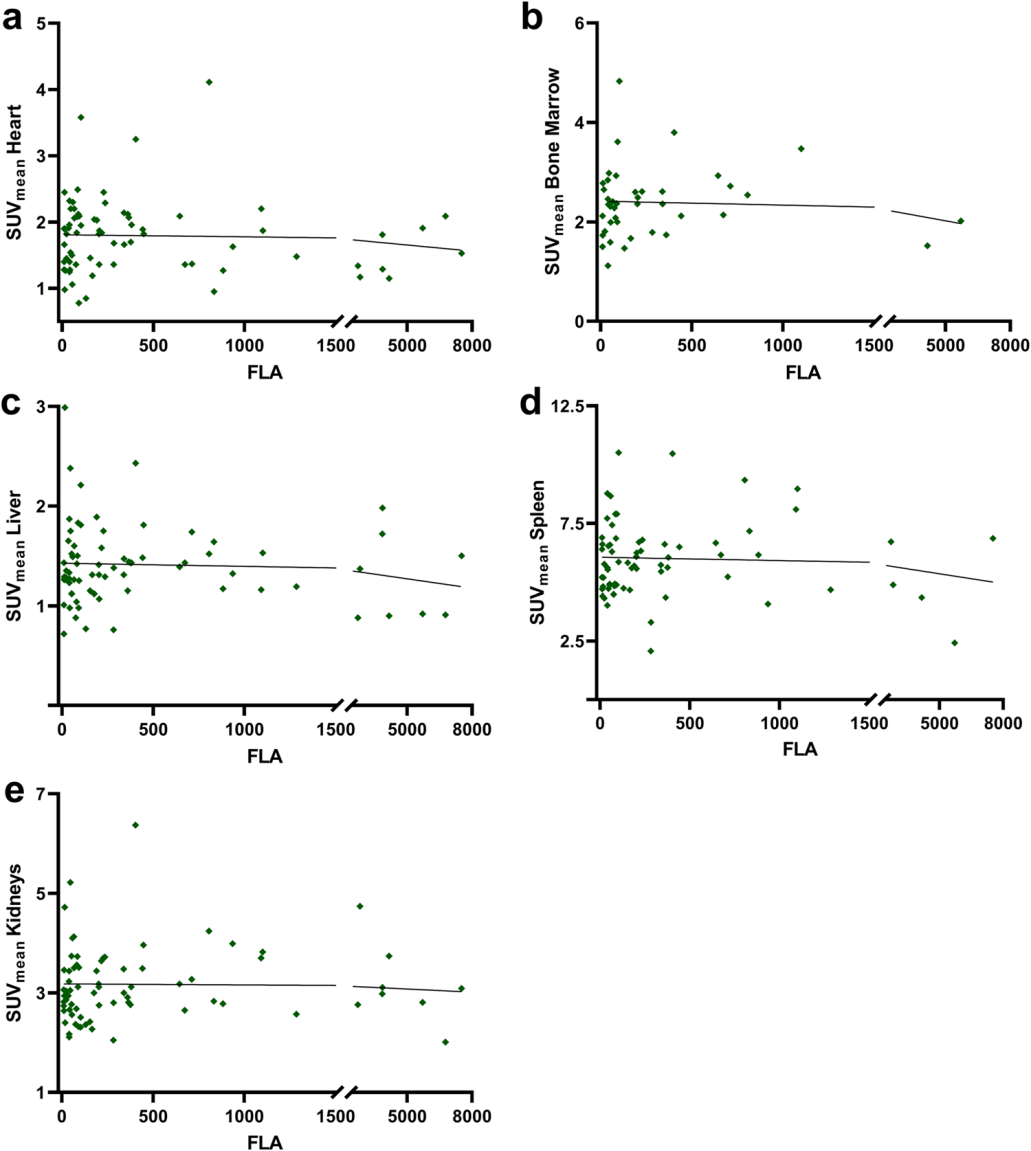
Scatter plots of lymphoma-derived fractional lymphoma activity (FLA) and mean standardized uptake values (SUV_mean_) in normal (heart (**a**), bone marrow (**b**), liver (**c**), spleen (**d**), kidneys (**e**)) Squares are partially overlaid. No significance was reached

**Fig. 2:**
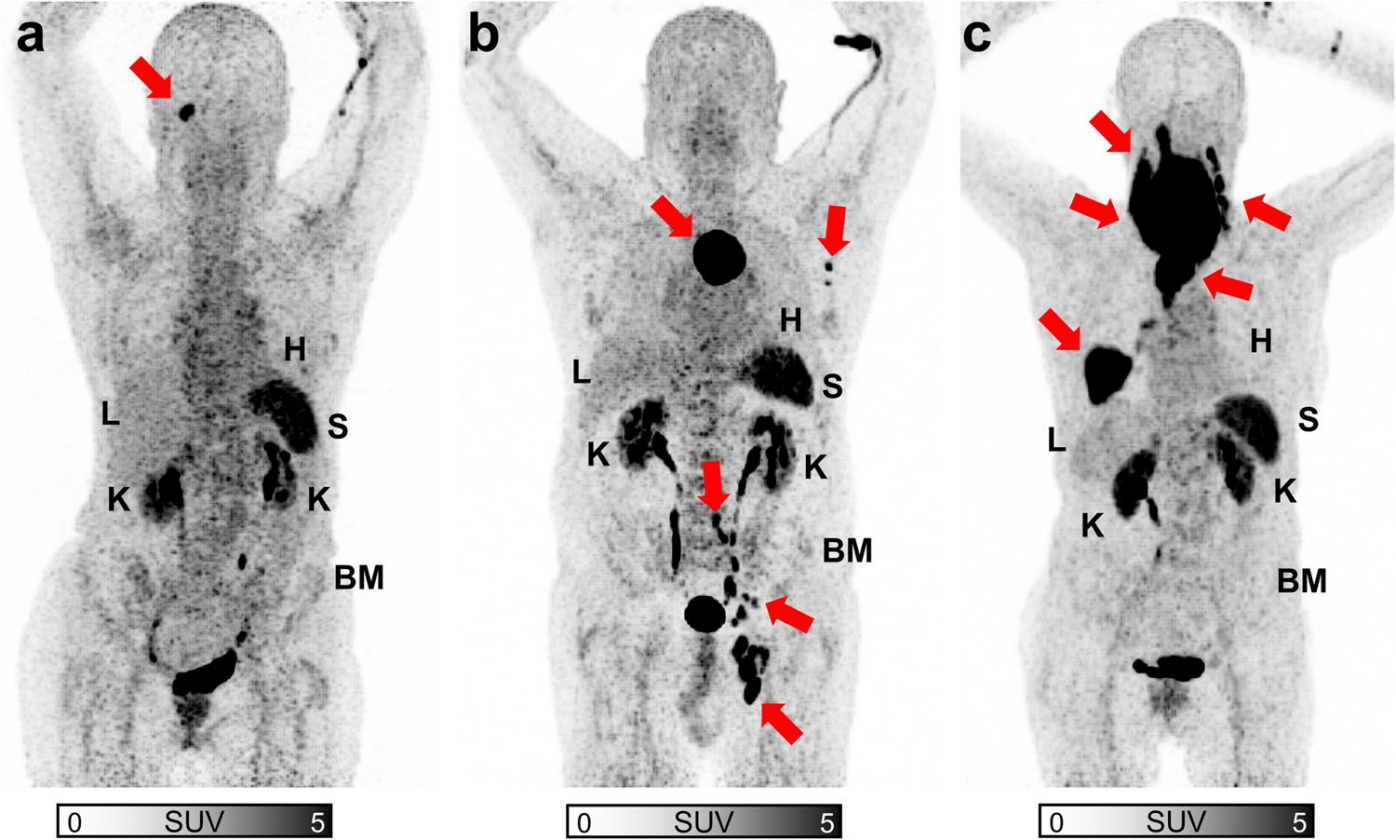
Maximum intensity projections of [^68^Ga]Ga-PentixaFor PET/CT in patients with low (**a** lymphoma volume, 1.7 cm^3^), intermediate (**b** lymphoma volume, 66.2 cm^3^) and high (**c** lymphoma volume, 361.5 cm^3^) lymphoma load. Red arrows indicate lymphoma manifestations. Visually, no obvious differences in normal organ uptake are apparent between different patients, supporting the notion that in patients with high lymphoma manifestation burden, uptake in normal organs does not drop. H = Heart, L = Liver, K = Kidney, S = Spleen, BM = bone marrow

A comprehensive summary of our correlative analyses is provided in Table 3. Figure 1 (along with Suppl. Figures 1, 2, and 3 (see ESM)) displays quantitative correlation plots between lymphoma manifestations and normal organ radiotracer uptake. We noted a trend towards significance for myocardial uptake and MZL manifestation for both SUV_peak_ (ρ = 0.20, *P* = 0.09; Suppl. Figure 1a (see ESM)), and SUV_max_ (ρ = 0.21, *P =* 0.07; Suppl. Figure 2a (see ESM)). Otherwise, no significant correlations were observed: SUV_max_ (ρ ≤ 0.19, *P* ≥ 0.16), SUV_peak_ (ρ ≤ 0.17, *P* ≥ 0.18), LV (ρ ≤ 0.13, *P* ≥ 0.27), and FLA (ρ ≤ 0.15, *P* ≥ 0.33). Visually, there was no apparent reduction in radiotracer uptake in normal organs with increasing lymphoma load, as shown in Fig. 2 in patients with low, intermediate, and high MZL burden.

**Table 3:** Spearman correlation (ρ) between normal organ radiotracer uptake and total MZL manifestation maximum and peak standardized uptake value (SUV_max_ and SUV_peak_), lymphoma volume (LV, in cm^3^), and fractional lymphoma activity (mean SUV*LV). A trend towards significance was only seen for myocardial uptake with lymphoma-derived SUV_max/peak_

			Lymphoma burden			
			SUV_max_	SUV_peak_	LV	FLA
Normal Organs	Heart	ρ	0.21	0.20	−0.08	0.00
		*P*	0.07	0.09	0.51	0.99
	Bone marrow	ρ	0.19	0.17	0.08	0.15
		*P*	0.23	0.30	0.60	0.33
	Liver	ρ	0.16	0.12	0.04	0.06
		*P*	0.18	0.31	0.76	0.61
	Spleen	ρ	0.17	0.14	−0.01	0.08
		*P*	0.16	0.27	0.91	0.51
	Kidneys	ρ	0.14	0.16	0.13	0.14
		*P*	0.23	0.18	0.27	0.24

## Discussion

Investigating a potential lymphoma-sink effect on CXCR4-targeted PET/CT for hematological malignancies, we evaluated a cohort of individuals diagnosed with MZL, which presented with a broad range of lymphoma load and *in-vivo* tracer uptake on PET. We did not observe relevant associations between normal organ uptake and lymphoma burden, thereby indicating that such a phenomenon may rather not be evident in MZL patients imaged with [^68^Ga]Ga-PentixaFor PET. Those findings may be of relevance, as recent reports provided evidence that CXCR4-targeted theranostics is particularly useful in patients with advanced blood cancers, especially for MZL [3, 4]. In this regard, CXCR4-targeted radioligand therapy (RLT) based on pretherapeutic PET/CT has already achieved outcome benefits in end-stage lymphoma patients [5, 18, 19], thereby rendering this theranostic concept favorable for the referring hemato-oncologist [6]. Based on our findings, dose to unaffected organs may not decrease in patients with increasing CXCR4-positive lymphoma load or with elevated PET signal. Thus, other aspects, such as intra- or interpatient variability of uptake in normal organs may be of more importance to consider dosimetry for CXCR4-RLT planning than the lymphoma load [15, 20].

Recent reports investigating a tumor-sink effect in the context of theranostic radiotracers provided dissimilar results, including PC imaged with 18F- or 68Ga-labeled PET radiotracers or in patients scheduled for fibroblast activation protein-inhibitor- or somatostatin receptor (SSTR)-directed imaging [8, 13–15, 21]. For instance, our group also evaluated this phenomenon in solid tumors after [^68^Ga]Ga-PentixaFor administration and reported on no impact of tumor burden uptake on normal organ uptake [9]. Those studies are of importance, as all of those radiotracers can be used in a theranostic approach, i.e., after having determined the presence of the target, treatment with ß^-^(minus)-emitting therapeutic equivalents can be conducted [22]. In this regard, higher activities could be administered for radiotracers harboring such a tumor-sink effect, which would then potentially result in higher doses to the tumor, but not to unaffected organs [21]. To date, however, investigations on a tumor-sink effect determined from baseline PET using theranostic tracers have been restricted to patients with solid tumors, including PC, neuroendocrine neoplasms, pancreatic cancer, lung or adrenocortical carcinoma [8, 9, 13–15, 21]. Recent years, however, have witnessed an increasing evaluation of CXCR4-directed imaging and therapy in particular for patients with hematological malignancies, thereby expanding this theranostic concept towards hemato-oncology [6]. While a potential lymphoma-sink effect has been observed on 2-[^18^F]FDG PET/CT in patients with DLBCL [10, 11], dedicated studies investigating a potential lymphoma-sink effect using the theranostic PET probe [^68^Ga]Ga-PentixaFor are still missing. To date, RLTs with the therapeutic equivalent [^177^Lu]Lu- or [^90^Y]Y-PentixaFor have been conducted based on pretherapeutic dosimetry to determine the appropriate amount of activity [5, 12, 19, 23, 24]. Based on our results, however, image findings on [^68^Ga]Ga-PentixaFor PET may not justify exceptional high treatment activities. Thus, while CXCR4-directed PET is required to determine the presence of the target *in-vivo* [6], those scans can rather not replace dosimetry prior to RLT.

In hemato-oncology, recent efforts also turned towards “cold” CXCR4 inhibitory therapies, e.g., by investigating CXCR4 antagonist IgG1 antibodies, which achieved substantial anti-lymphoma effects by disrupting SDF1 pathways [25]. Preclinical models showed relevant benefit in multiple myeloma, acute myeloid leukemia or Hodgkin Lymphoma [25]. Of note, in all of those malignancies, [^68^Ga]Ga-PentixaFor has provided evidence on visualizing chemokine receptor expression in sites of disease [7, 26, 27]. As such, given those recent developments of CXCR4-directed, non-radiolabeled drugs [25], our findings on a missing lymphoma-sink effect on PET may be of relevance not only for “hot,” but also for “cold” CXCR4 inhibitory therapies, including dosing studies.

This investigation has limitations, e.g., its retrospective design and small number of subjects. Also, no additional weighting was made for different volumes of segmented lymphoma lesions when calculating SUV values within the same patient. Additionally, a potential lymphoma sink effect may be underestimated because only a small number of patients in our study had very high LV or FLA. Moreover, due to the retrospective nature of this study, we were not able to provide data on an *ex-vivo* blood count analysis, which might have offered further insights on a potential lymphoma sink effect by measuring blood pool activity. Furthermore, while previous investigations focusing on other theranostic radiotracers have mainly applied relative thresholds for tumor delineation [13, 15], a recent investigation also used a fixed intra-individual threshold [8], e.g., based on the liver uptake. As this approach might allow for a more comparable tumor load delineation between scans, future studies may also conduct a respective re-analysis, preferably in a larger cohort with a broader range of LV. Last, we focused on MZL, as relative to others, this type of hematological malignancy has been well characterized by CXCR4-directed imaging including superiority to standard diagnostic work-up [3, 4], indicative for a more widespread use in patients with MZL in the near-term future. However, [^68^Ga]Ga-PentixaFor has also provided reliable information on the current chemokine receptor status in other hemato-oncological entities [4]. As such, future studies may also evaluate a potential lymphoma-sink effect in other subtypes, in particular in those which have already benefitted from CXCR4-RLT, including T-cell lymphoma [5].

## Conclusions

We observed no relevant associations between normal organ uptake and CXCR4-positive lymphoma burden in patients with MZL studied with [^68^Ga]Ga-PentixaFor PET/CT. As such, relative to other theranostic radiotracers used for imaging and therapy of solid tumors, a lymphoma-sink effect may rather not be evident in MZL. Those findings may be of relevance in a theranostic setting, as recent reports provided evidence that CXCR4-targeted RLT is particularly useful in patients with advanced blood cancers. In addition, the herein described missing lymphoma-sink effect may also be of relevance for dosing studies using novel CXCR4-inhibitory “cold” drugs.

## Supplementary Information

ESM 1

## Data Availability

The datasets generated and analyzed during the current study are available from the corresponding author upon reasonable request.
