# Supplementary material for: Lymphoma-Sink Effect in Marginal Zone Lymphoma Based on CXCR4-Targeted Molecular Imaging
Source: Mol Imaging Biol. 2023 Jun 7;25(4):758–64. doi: 10.1007/s11307-023-01830-9 (PMC10333359; doi:10.1007/s11307-023-01830-9)

Electronic Supplementary Material

Lymphoma-Sink Effect in Marginal Zone Lymphoma based on CXCR4-Targeted Molecular Imaging

Journal: Molecular Imaging and Biology

Aleksander Kosmala^1^, Simone Seifert^1^, Simone Schneid^1^, Niklas Dreher^1^, Takahiro Higuchi^1,2^, Alexander Weich^3^, Sebastian E. Serfling^1^, Philipp E. Hartrampf^1^, Herrmann Einsele^3^, Andreas K. Buck^1^, Max S. Topp^3^, Johannes Duell^3,*^, and Rudolf A. Werner^1,4,*^

^1^ Department of Nuclear Medicine, University Hospital Würzburg, Würzburg, Germany;

^2^ Faculty of Medicine, Dentistry and Pharmaceutical Sciences, Okayama University, Okayama, Japan;

^3^ Department of Internal Medicine II, University Hospital Würzburg, Würzburg, Germany;

^4^ Johns Hopkins School of Medicine, The Russell H Morgan Department of Radiology and Radiological Sciences, Baltimore, MD, United States;

^*^ contributed equally

*Correspondence:*

Rudolf A. Werner, MD

Department of Nuclear Medicine

University Hospital Würzburg

Oberdürrbacher Strasse 6

97080 Würzburg, Germany

Phone: +49 931 201 35001

E-mail: werner_r1@ukw.de

**Supplementary Fig. 1** Scatter plots of peak standardized uptake values (SUV_peak_) derived from lymphoma manifestations and mean standardized uptake values (SUV_mean_) in normal organs (heart, **a**; bone marrow, **b**; liver, **c**; spleen, **d**; kidneys, **e**). Squares are partially overlaid. No significance was reached.


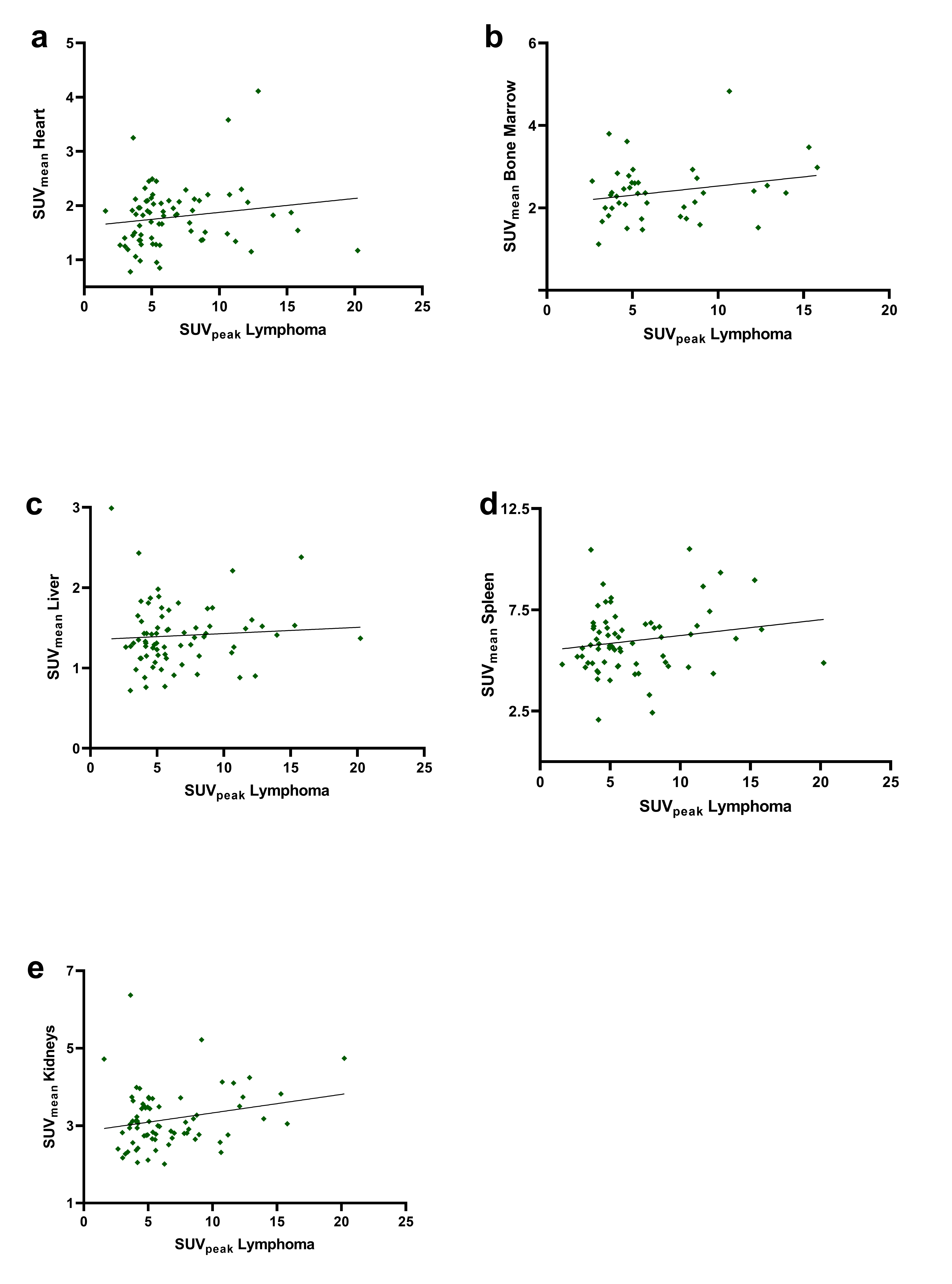


**Supplementary Fig. 2** Scatter plots of maximum standardized uptake values (SUV_max_) derived from lymphoma manifestations and mean standardized uptake values (SUV_mean_) in normal organs (heart, **a**; bone marrow, **b**; liver, **c**; spleen, **d**; kidneys, **e**). Squares are partially overlaid. No significance was reached.


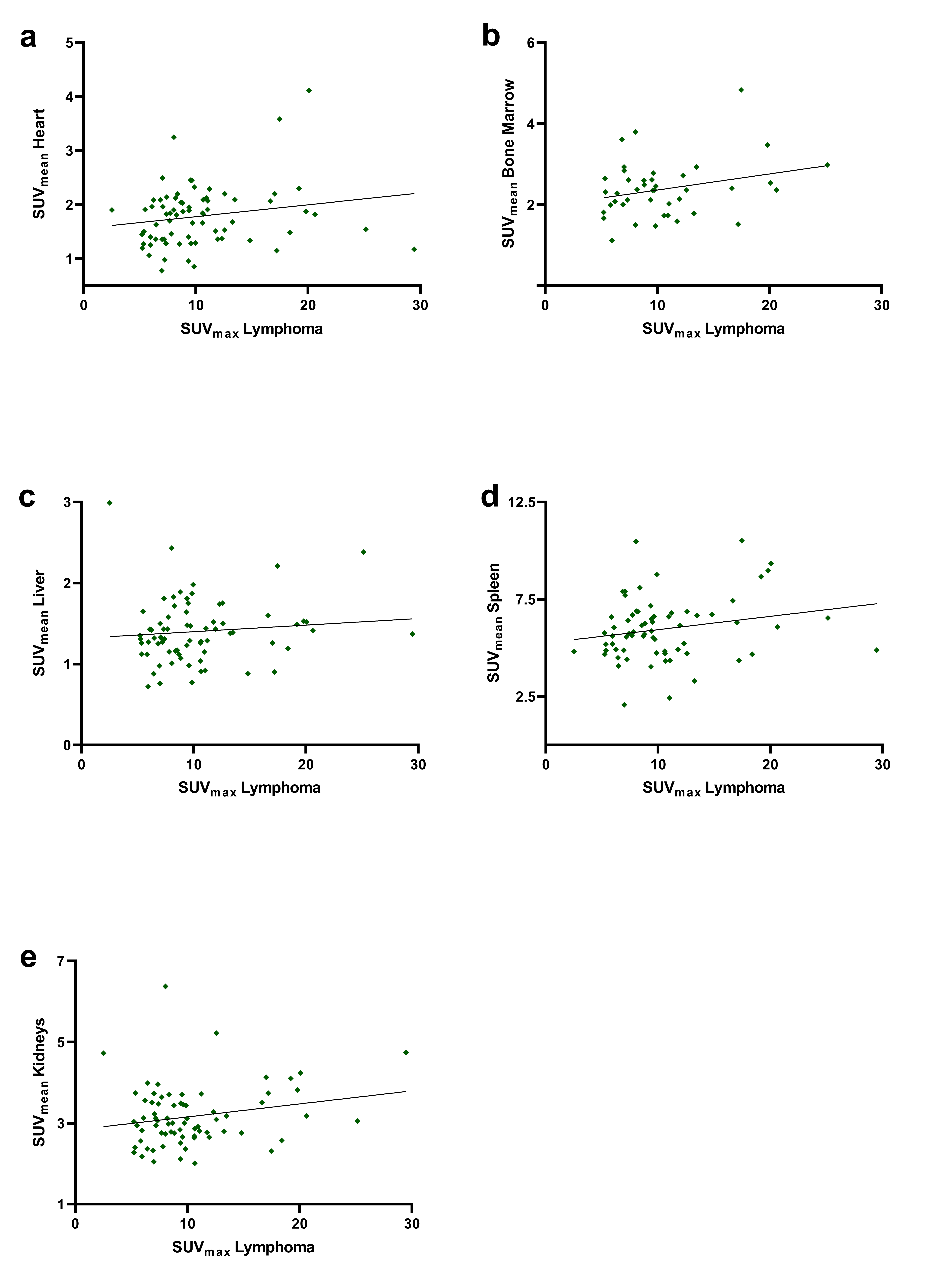


**Supplementary Fig. 3** Scatter plots of lymphoma volume (in cm^3^) and mean standardized uptake values (SUV_mean_) in normal organs (heart, a; bone marrow, b; liver, c; spleen, d; kidneys, e). Squares are partially overlaid. No significance was reached.


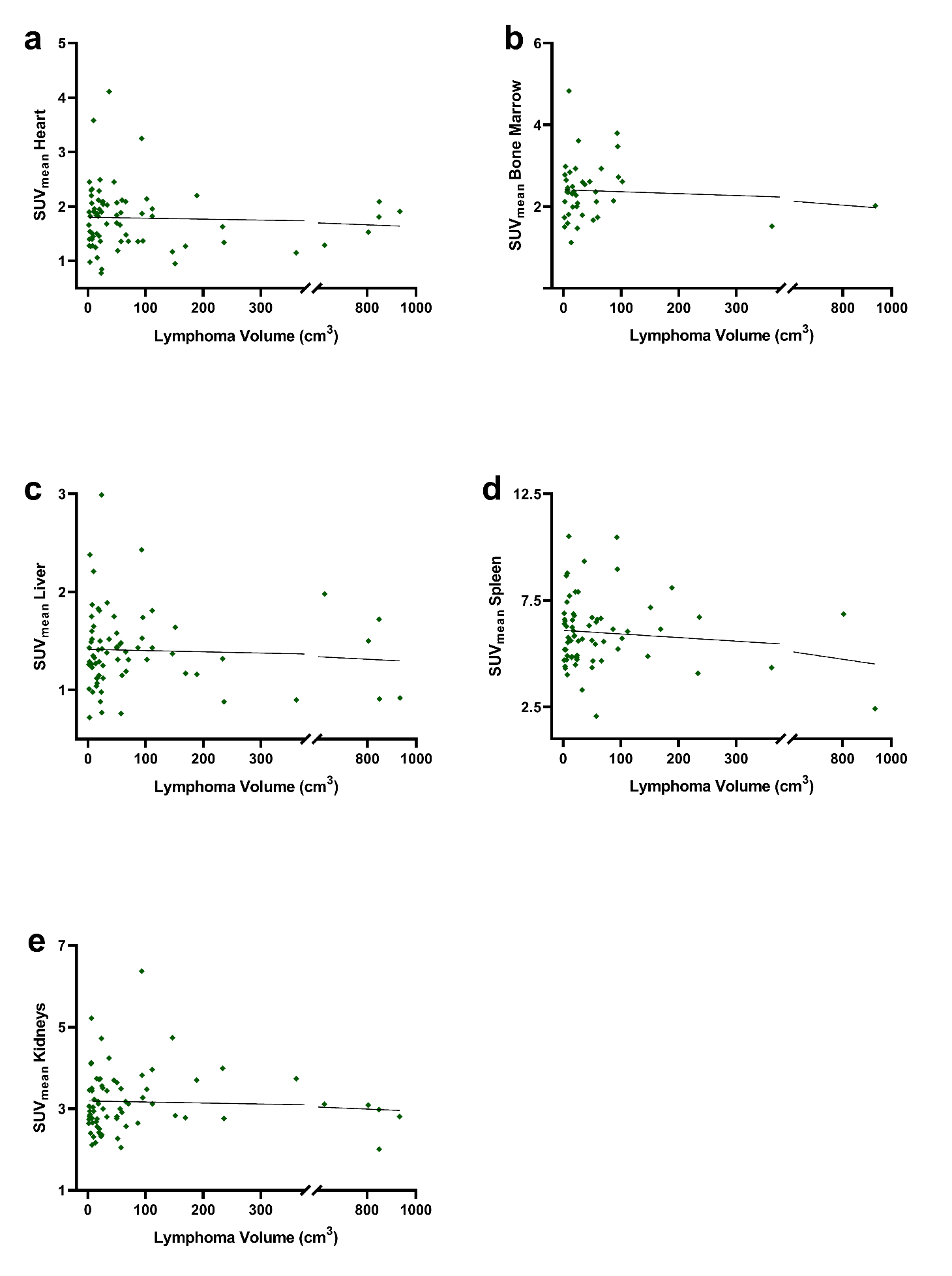

Supplement: Supplementary file 1 — ESM 1 [file 11307_2023_1830_MOESM1_ESM.docx]
